# Free-roaming dogs limit habitat use of giant pandas in nature reserves

**DOI:** 10.1038/s41598-020-66755-7

**Published:** 2020-06-24

**Authors:** Ramana Callan, Jacob R. Owens, Wenlei Bi, Benjamin Kilham, Xia Yan, Dunwu Qi, Rong Hou, James R. Spotila, Zhihe Zhang

**Affiliations:** 1 Sichuan Key Laboratory of Conservation Biology for Endangered Wildlife, Chengdu Research Base of Giant Panda Breeding, Chengdu, Sichuan 610081 P.R. China; 2 Drexel University, Philadelphia, PA 19104 USA; 3 Kilham Bear Center, Lyme, NH 03768 USA

**Keywords:** Ecology, Zoology, Ecology

## Abstract

Giant pandas (*Ailuropoda melanoleuca*) were historically hunted using dogs and are currently threatened by free-roaming dogs and their associated diseases. To better understand the spatial magnitude of this threat, we used a GIS approach to investigate edge effects of dogs on giant panda habitat. We first examined two nature reserves with contrasting free-roaming dog populations: Liziping, with many dogs (~0.44/km^2^), and Daxiangling, with few dogs (~0.14/km^2^). Spatial analysis indicated that giant pandas at Liziping (but not Daxiangling) showed a shift in habitat use away from populated areas consistent with a risk response to the foray distance of free-roaming dogs (10.9 km path-distance). Most giant panda locations (86%) from the 2014 census in Liziping were clustered around remote “dog-free zones.” Expanding this analysis across the entire giant panda range revealed that 40% of panda habitat is within the foray distance of dogs. Our assessment will inform dog control programs including monitoring, education, veterinary care, and other measures. We recommend that reserves designated for the release of translocated pandas receive priority consideration for dog control efforts. Only by understanding and managing complex interactions between humans, domestic animals, and wild animals can we sustain natural systems in a world increasingly dominated by humans.

## Introduction

Nature reserves are primary sanctuaries for threatened species but are vulnerable to anthropogenic pressures from outside their borders^1,2^. Edge effects, resulting in changes to species abundance and distribution, frequently occur at the administrative borders of reserves^3,4^. Lower abundance near edges is indicative of negative edge effects at the population level^5^ and mammalian carnivores are especially sensitive to this global threat^3^. Edge effects are not confined to a few hundred meters, as was previously thought, but can persist 10 km from a habitat edge, thus impacting the conservation of biodiversity at landscape scales^6^. However, this knowledge has failed to translate into management reform for even some of our most iconic threatened species^4,7^. Due to a complexity of socioecological factors, conservation efforts, such as reserve planning and corridor placement, have consistently failed to incorporate mitigation techniques for addressing the role reserve edges play as population sinks for species of concern.

Species interactions such as competition, disease transmission, and predation are altered at edges^8^. Collectively, these factors favor species that thrive in disturbed environments (such as invasive and generalist species) at the expense of area sensitive native species^9^. The link between edge effects and invasive species has been well documented^10^. Edges act as gateways for invasive species that can facilitate dispersal into the interior^9^.

One such invasive species is the domestic dog (*Canis lupus familiaris*). The global dog population is estimated to exceed 700 million, making dogs the most abundant carnivore worldwide^11^. Approximately 75% of these dogs are free-roaming^12^. When free-roaming dogs are associated with rural residences bordering nature reserves, conflicts with wildlife become inevitable^13–15^. Feral and free-roaming dogs have not received as much attention as feral cats in the scientific literature^16^. However, dogs can have profound effects on wildlife. Dogs predate wildlife^13,17,18^, spread disease^19,20^, harass wildlife^21^, cause impacted species to limit their habitat use^22,23^ and reduce breeding success^24^. Dogs have even been implicated in the extinction of 11 species and are considered a potential threat to over 188 additional species of concern^25^.

Predation, harassment and disease transmission by dogs can have large-scale edge effects in both fragmented habitats^26^ and protected nature reserves^13^. Although landscape level edge effects due to feral and free-roaming dogs have been reported for a handful of species^15,23,27^, there is an urgent need for basic guidelines outlining how to address this problem. How do we incorporate the edge effects of dogs into the management of protected areas to mitigate impacts on threatened species? Here, we provide strategies for how to measure potential impacts at a landscape scale and outline how to incorporate feral-dog management into the landscape level planning of species conservation using the giant panda as an example.

Giant pandas (*Ailuropoda melanoleuca*) require a minimum habitat size of 114 km^2^ to enable long-term persistence of local populations^28^. Most nature reserves set aside for giant pandas exceed this area requirement. However, nature reserves are often closely connected to human settlements. The Wolong Nature Reserve is the most famous of the 67 panda reserves in China. It protects approximately 2,000 km^2^ of land but contains over 5,000 people in 1,436 households^29^. The human residents and their domestic animals directly and indirectly affect giant pandas and the rate of habitat decline actually increased after the reserve was expanded in 1975^30^. When edge effects are accounted for, the suitable habitat within these reserves may fall well below the minimum area requirement for giant pandas. Incorporating edge effects when determining effective habitat area (effective habitat area = baseline habitat minus edge effects) is essential for the conservation and management of giant pandas. In addition, edge effects should be considered when assessing suitable sites to release captive-reared or translocated individuals. Many reserves with extant giant panda populations may already be at carrying capacity for the available effective habitat.

As with most bear species, giant pandas were historically hunted using dogs^31^ and are still harassed and chased by dogs in nature (personal observation). Giant pandas are susceptible to mortality from canine distemper virus and canine coronavirus^32^. Antibodies for both of these viruses were documented 25 years ago in free-roaming dogs and giant pandas within the Wolong Nature Reserve in Sichuan, China^33^. A more recent study conducted by our lab in Liziping Nature Reserve revealed that 21% of village dogs surveyed had positive antibody titers for at least one of four viruses potentially lethal to giant pandas: canine distemper, parvovirus, rotavirus and/or rabies^34^. In addition, 67% of village dogs were positive for gastrointestinal parasites, including two species known to infect giant pandas: *Ancylostoma caninum* and *Strongyloides* sp.^34^. Despite these facts, resource managers do not consider free-roaming dogs to be a major threat to wild giant pandas. There are no comprehensive plans in place to remove free-roaming and feral dogs from nature reserves or to control dog populations in local villages within the giant panda range.

Although considerably smaller than bears, dogs are effective hunters of many bear species. Dogs hunt in packs and can move quickly through the dense vegetation used by giant pandas. Harassment by dogs can lead to increased stress and energetically costly behavior for many species^21^. Even if dogs do not kill pandas directly, dog attacks can result in wounds that lead to severe infections or cause stress related myopathies that ultimately result in mortality. Dogs may pose an even greater threat to giant panda cubs, which are particularly vulnerable as the most altricial of all bear species. Based on these risks, we predict that the presence of dogs will limit habitat use by giant pandas and reduce the available effective habitat in nature reserves throughout the range of the giant panda. Giant pandas within the “dog zone” will likely incur additional costs to reproduction and survival due to stress and altered foraging behavior, making the “dog zone” either a sink habitat or an ecological trap.

Some previous models of giant panda habitat suitability have recognized that pandas avoid human-dominated landscapes. These models incorporated distance from roads and residential areas in predicting habitat use^28,35–38^. However, these models are potentially deficient where large populations of free-roaming dogs have access to giant panda habitat. The edge effect of free-roaming dogs may be considerably greater than the distances used in these studies.

Proximity to human residences is the most important factor in predicting rural dog distribution^39^. Foray distances away from human residences differ considerably amongst movement studies of free-roaming and feral dogs (Table 1) indicating a need for more comprehensive studies. Giant pandas (and wildlife in general) are exposed to the threat of free-roaming dogs within this foray distance, even deep within nature reserves. In 2016 and 2017, we observed free-roaming dogs from local villages, alone and in packs of up to 4 individuals, traveling in and out of the Liziping Nature Reserve^34^. These observations are consistent with previous studies of group size of feral and free-roaming dogs in other ecosystems^40^. Group size of village dogs and a transition to more “wolf-like” behavior are likely driven by factors such as the amount of provisioning provided by humans, the age, gender and size of the dogs, and whether they routinely accompany their owners into wilderness areas^41^.

**Table 1:** Published maximum distances traveled by feral and free-roaming dogs.

Source	Maximum Distance
Meek, 1999^57^	30.0 km (Euclidean distance)
Sepúlveda, *et al*., 2015^39^	4.3 km (Euclidean distance)
Scott & Causey, 1973^58^	8.2 km (Euclidean distance)
Butler, Du Toit, & Bingham, 2004^13^	6.0 km (Euclidean distance)
This study	10.9 km (path-distance)

Given that nature reserves will vary in their susceptibility to free-roaming dog populations based on the local density of human residences and their spatial relationship to the protected wildlife habitat, we sought to model the potential edge effect of known populations of free-roaming dogs on giant panda habitat at a landscape and then regional scale. In our study we asked the question: How can knowledge of the spatial extent of the free-roaming dog threat be used to inform management for giant pandas at a regional scale? We first determined the distance that a free-roaming dog will travel in giant panda habitat (foray distance) using a path-distance tool and direct observations of dogs traveling away from villages into a local giant panda nature reserve. We then compared the edge effect of free-roaming dogs on giant panda habitat use in two different nature reserves that differed in their free-roaming dog populations. Once we had tested the utility of this approach, we expanded the scope of the study to a regional scale and assessed the potential spatial threat of free-roaming dogs across all 67 giant panda nature reserves. Used sequentially, these steps provide a model approach for incorporating the spatial extent and magnitude of edge effects of free-roaming dogs into species-oriented management plans at regional scales.

## Materials and Methods

### Study area

**Figure 1:**
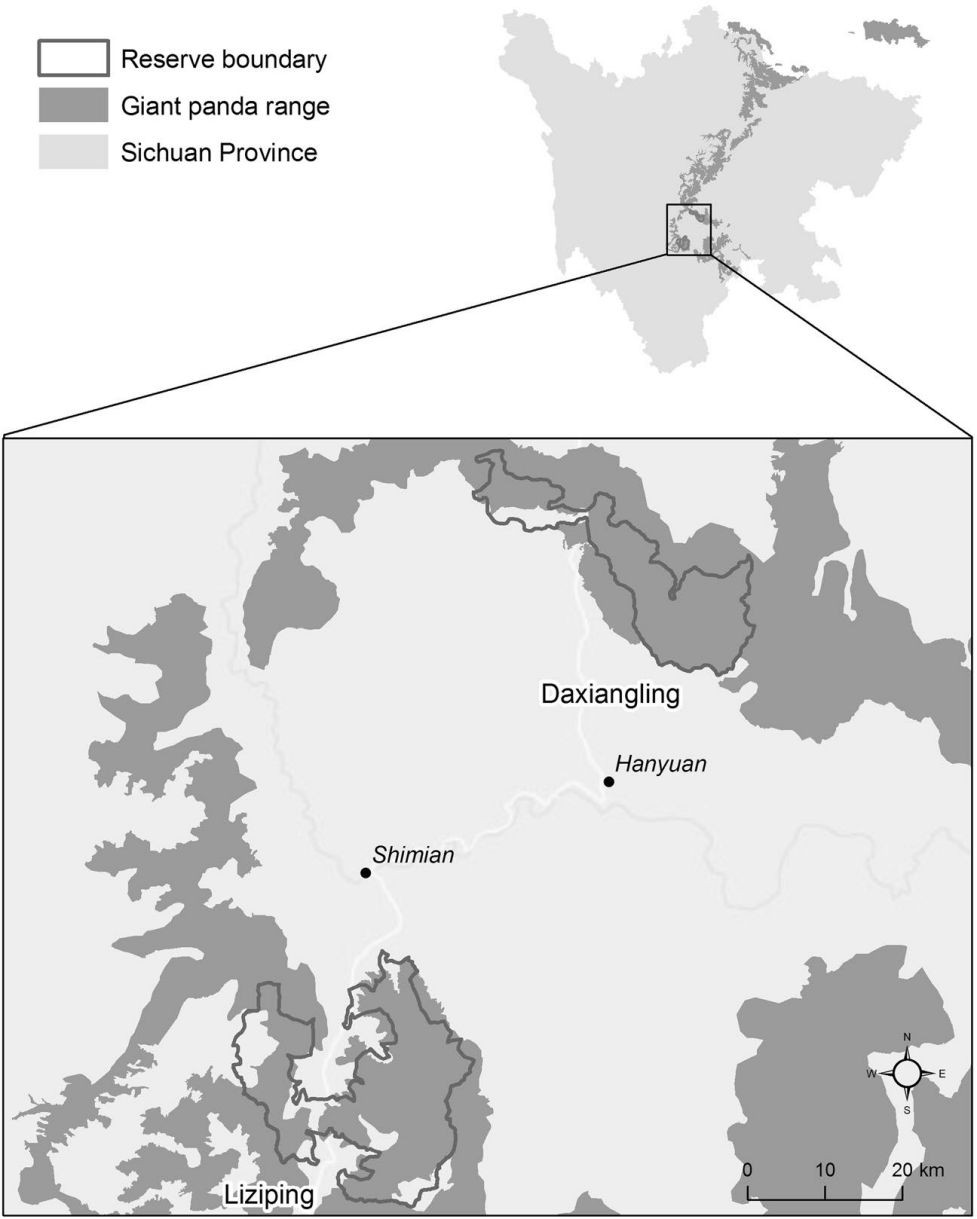
Study area map showing Liziping and Daxiangling Nature Reserves in Sichuan Province, China. Map produced using ArcGIS 10.6.1: https://desktop.arcgis.com/en/.

Liziping and Daxiangling Nature Reserves are important locations for giant panda conservation. Both reserves are located in Sichuan Province, China, 102°-103°E, 28°-29°N (Fig. 1). These reserves cover 479 km^2^ and 295 km^2^, respectively; however, Daxiangling is adjacent to Long Canggou National Park, as well as the Wawushan Nature Reserve, making the contiguous protected habitat area much larger. Liziping is the present site for giant panda translocations and releases into the wild^42^. The landscape in both reserves is rugged, comprised of ridges and narrow valleys, although Daxiangling’s terrain is somewhat gentler. Elevation ranges from 1,330–4,800 m in Liziping and 1,300–3,400 m in Daxiangling. The average annual temperature is 14 °C in Liziping and 16 °C in Daxiangling. The forests in both reserves are primarily broad-leaf evergreen at lower elevations and transition into coniferous forest around 2,400 m. Dominant bamboo species in the understory at Liziping are *Yushania lineolata* (at 2,000–2,600 m) and *Arundinaria spanostachya* (above 2,500 m). At Daxiangling, *Chimonobambusa szechuanensis* is abundant below 2,400 m and *Arundinaria faberi* is found at 2,400–3,200 m.

Comprehensive surveys by our laboratory of the six village groups surrounding Liziping in 2017 found that 212 of 334 owned dogs in those villages were free-roaming (64%). In contrast, a concurrent survey of a large village group (including 11 villages) proximate to Daxiangling found that only 42 of 110 owned dogs were free-roaming (38%). Thus, the threat of free-roaming dogs was far greater in Liziping than in Daxiangling.

### Path-distance from residences

We digitized residences within 8 km of both nature reserves from aerial imagery (2.5 m resolution Spot imagery from 2015 provided by ArcGIS Online) at a 1:10,000 scale. We classified areas with housing density greater than 6.25 houses per km^2^ as “populated areas.” We then used these data and a Digital Elevation Model (ASTER Global DEM, a product of METI and NASA) to run the path-distance tool in ArcGIS. This created a continuous surface representing distance (combined vertical and horizontal) away from populated areas. We incorporated vertical as well as horizontal distance because the terrain in our study area was very steep and likely to influence access by free-roaming dogs. This process essentially created a series of buffers that account for terrain complexity.

### Dog-free zones

The maximum distance we observed for free-roaming dogs in Liziping, based on direct field observations and preliminary results of an ongoing camera trap study, was 10.9 km (path-distance). This distance is much greater than the 1.92 km distance estimated by previous studies as defining the impact of human residences on giant panda habitat use^36,43^. Assuming that dogs can travel 10.9 km (path-distance) from their residence, we extracted the area within this distance to map potentially “dog-free” zones for giant pandas within both nature reserves. We assumed that all residences had the potential to house free-roaming dogs as the ability to completely census each household was beyond the scope of this study.

### Giant panda habitat and survey data

Although over 23 studies have been published on giant panda habitat selection^44^, there is currently no broadly accepted range-wide model of suitable giant panda habitat. Therefore, for the purposes of this study, we used the IUCN designated extant giant panda range^45^ to represent baseline available habitat for giant pandas within and surrounding each reserve. This dataset was readily available for the entire range of the giant panda in China and allowed for repeatability. Henceforth, we refer to the IUCN giant panda range dataset as “giant panda habitat” when referring to local spatial scales of habitat use and availability. We intentionally made the analysis for this study straightforward using readily available datasets so that resource managers can easily repeat the process.

Location data for giant pandas were from the Fourth National Giant Panda Census^46^ in Sichuan Province. This survey was carried out from 2011 to 2013. Giant panda occurrence locations were determined by the presence of feces, footprints and foraging traces. We only had access to census data from the two reserves that we worked in, Liziping and Daxiangling.

### Giant panda habitat use

To assess whether giant pandas were using habitat relative to its availability, we first generated 100 random locations within the baseline giant panda habitat in each nature reserve (Liziping and Daxiangling) to represent available habitat. We then compared the distribution of giant panda locations from the giant panda survey data to the distribution of available habitat (as a function of path-distance from a residence).

### Extrapolation to range-wide impacts

Using Liziping and Daxiangling Nature Reserves as case studies, we extended our spatial analysis to incorporate the entire extant giant panda range (including all 67 giant panda nature reserves). Given the vast extent of the giant panda range, we digitized populated areas at a scale of 1:40,000 within a buffer of 15 km (instead of at a scale of 1:10,000 as was done for the case studies). Again, we used Spot imagery from 2015 provided by ArcGIS Online. The same steps were used to model path-distance away from populated areas as were employed in the reserve level analysis described above. The edge effect of free-roaming dogs (10.9 km path-distance) was then extracted from the extant giant panda range to represent effective giant panda range.

We prioritized the 67 giant panda nature reserves for dog control programs by calculating the total area of panda habitat within 10.9 km path-distance of human residences within each nature reserve. Nature reserves were categorized as having a low dog threat if the total area of giant panda habitat within the “dog zone” was <50 km^2^, as moderate if the total area was <165 km^2^, and as high if the total area was > 165 km^2^). The break points were selected using the Jenks optimization method which maximizes the variance between classes. We used ArcGIS 10.6.1 for all analyses and the production of all maps.

## Results

### Path-distance from residences

**Figure 2:**
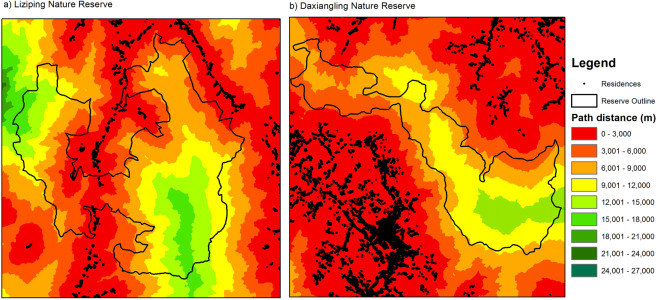
Path-distance from populated areas in Liziping and Daxiangling Nature Reserves. “Populated areas” are defined as areas with housing density greater than 6.25 houses per km^2^ (shown in black on the map). “Path distance” is the combined vertical and horizontal distance (in meters) that a free-roaming dog would need to travel away from a populated area to reach a certain point on the landscape. Red areas are within 3 km of a populated area while green areas are 12–18 km from a populated area (based on path distance). Map produced using ArcGIS 10.6.1: https://desktop.arcgis.com/en/.

We recorded 8,400 residences within an 8 km radius of Liziping Nature Reserve and 44,100 residences within an 8 km radius of Daxiangling Nature Reserve (Fig. 2). These residences were mostly distributed at lower elevations along major roads and rivers. Path-distance models indicated that the majority of area within both reserves was within 12 km of a residence, and none of the area within either reserve was more than 18 km from a residence (path-distance, not Euclidean distance).

### Dog-free zones

**Figure 3:**
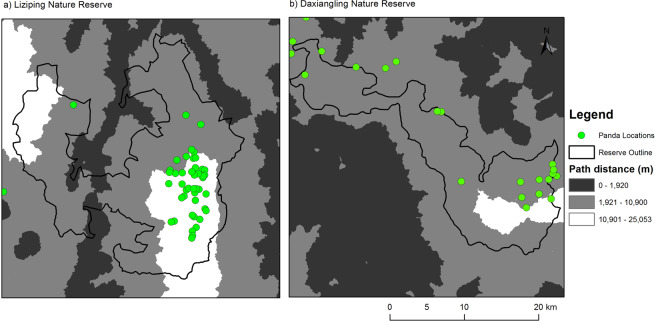
Path-distance from populated areas within the human impact zone (1,920 m shown in dark gray) and the maximum foray distance (10,900 m shown in light gray) of free-roaming dogs in Liziping (with panda locations from 2014 Fourth National Giant Panda Census). White areas indicate “dog-free zones”. However, there were no dogs reported in Daxiangling Nature Reserve. Map produced using ArcGIS 10.6.1: https://desktop.arcgis.com/en/.

None of the giant panda locations from the 2014 Fourth National Giant Panda Census were closer than 2 km to a populated area in either reserve (Fig. 3). In Liziping, where free-roaming and feral dogs numbered in the hundreds (see Methods), giant panda locations were clumped in the “dog-free zone” at a distance greater than 10.9 km from a populated area (of the 46 giant panda locations, 38 were more than 10.9 km from a populated area). At Daxiangling, where there were few free-roaming dogs in villages and no dogs reported in the reserve, most of the giant panda locations were between 2 and 12 km from a populated area and did not show the clumped pattern of spatial distribution seen in Liziping.

**Figure 4:**
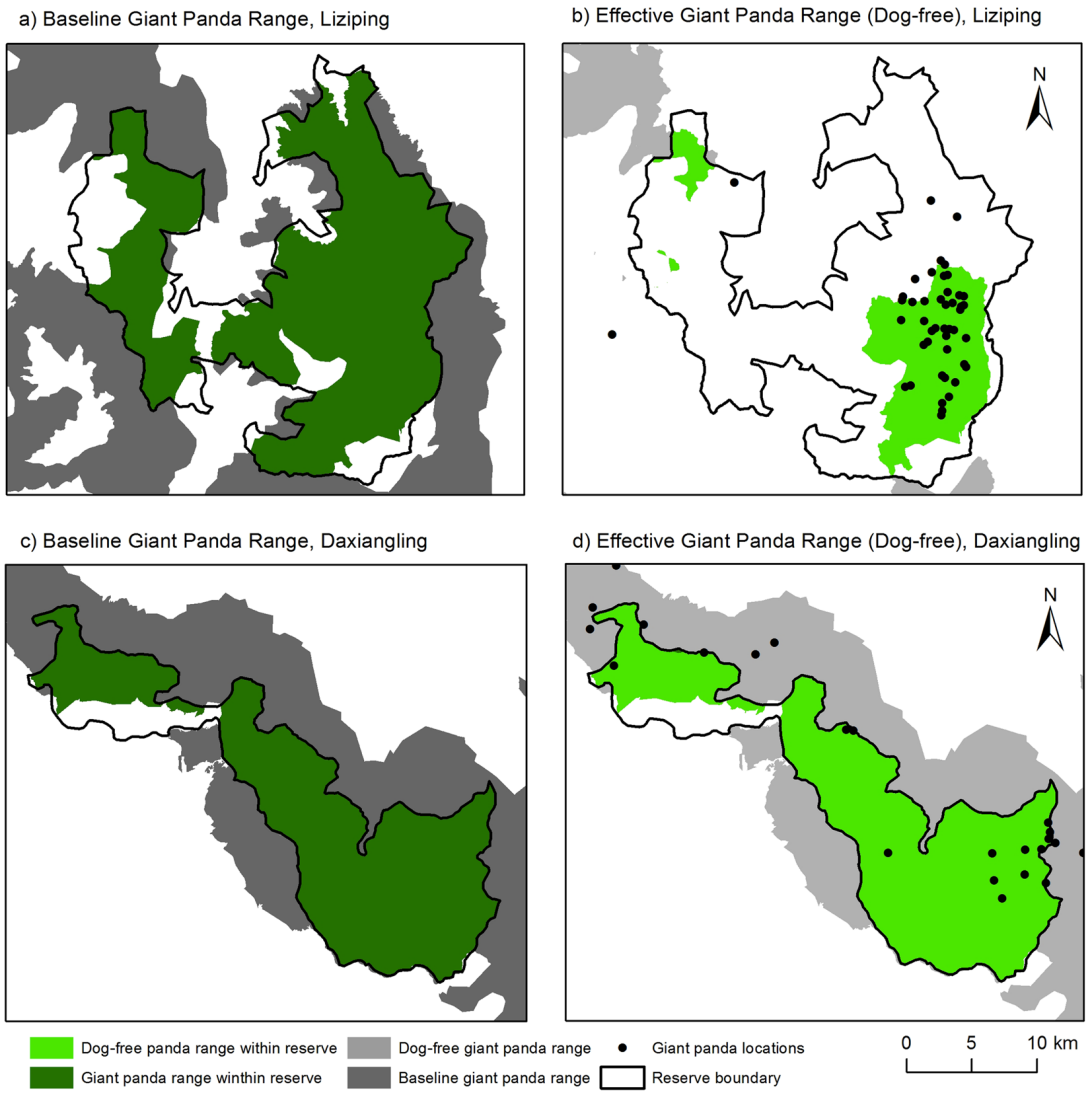
Baseline and effective giant panda habitat for Liziping and Daxiangling Nature Reserves. (**a**) Baseline protected and unprotected giant panda habitat in and surrounding Liziping. (**b**) Effective giant panda habitat at Liziping when the influence of free-roaming dogs is incorporated (with panda locations from 2014 Fourth National Giant Panda Census). (**c**) Baseline and effective giant panda habitat in and surrounding Daxiangling. d) Effective giant panda habitat at Daxiangling (with panda locations from 2014 Fourth National Giant Panda Census). Map produced using ArcGIS 10.6.1: https://desktop.arcgis.com/en/.

To address the edge effect of free-roaming dogs, we mapped the effective panda habitat for Liziping and Daxiangling by extracting habitat within the 10.9 km average foray distance of free-roaming dogs (Fig. 4). Protected baseline giant panda habitat in both reserves (Table 2) was above the 114 km^2^ threshold identified as the minimum habitat size required for giant pandas^28^. When the effect of free-roaming dogs at Liziping was accounted for, effective habitat area was reduced from 385.2 km^2^ to 108.7 km^2^. Perhaps equally alarming is the impact of edge effects on the giant panda habitat outside Liziping Nature Reserve (shown in gray in Fig. 4a,b).

**Table 2:** Difference between baseline protected area (within the reserve) and effective protected area of giant panda habitat for two nature reserves.

	Baseline Giant Panda Habitat within each reserve	Effective Giant Panda Habitat I (human impact)	Effective Giant Panda Habitat II (human and dog impact)
Liziping	385.2 km^2^	380.3 km^2^	108.7 km^2^
Daxiangling	271.2 km^2^	271.1 km^2^	63.9 km^2^*

Areas provided were based on the impact of humans alone (scenario I: within the reserve and greater than 1.9 km path-distance from a populated area) and human and free-roaming dogs together (scenario II: within the reserve and greater than 10.9 km path-distance from a populated area).

*Although surveys indicate no current dog problem in Daxiangling, this is the effective area that would result from a future free-roaming dog population in Daxiangling.

**Figure 5:**
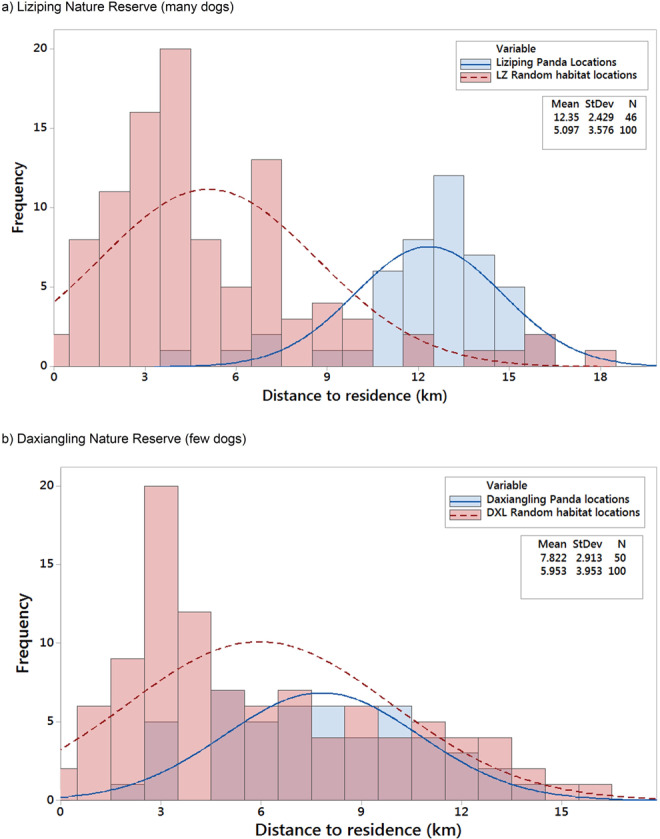
Histogram showing available habitat versus habitat used by giant pandas as a function of path-distance from a populated area in two nature reserves in Sichuan Province, China. (**a**) Liziping (many dogs) and (**b**) Daxiangling (few dogs). Means and standard deviations represent the average path-distance from a populated area for available habitat (shown in red bars and based on 100 random locations within suitable habitat in each reserve) and used habitat (shown in blue bars and based on 46 and 50 giant panda locations from the 2014 Fourth National Giant Panda Census).

Humans without free-roaming dogs (impact distance of 1.92 km^35^) appear to have a minimal edge effect on giant panda habitat within the nature reserves (as seen in Daxiangling). In contrast, free-roaming dogs (impact distance of 10.9 km) have the potential to drastically reduce effective habitat area (as seen in Liziping). In the case of Liziping, edge effects from free-roaming dogs could reduce effective habitat below the 114 km^2^ threshold required for long-term population viability. In contrast, the effective giant panda habitat for Daxiangling (271.1 km^2^), modeled based on the effect of humans without free-roaming dogs, was not reduced by edge effects as the villages were more than 2 km from the reserve boundary. However, if the dog population grows at Daxiangling, and dogs enter the reserve, we predict that 76% of the current habitat will be affected (Table 2).

### Use versus availability

By comparing habitat use to habitat availability, we found that at Liziping the habitat use of giant pandas (as a function of path-distance from populated areas) was shifted approximately 7 km farther away from populated areas than would be expected given the distribution of available panda habitat (Fig. 5). This shift in distance is two standard deviations farther away than the mean distance to available protected panda habitat (5.1 km vs. 12.4 km). Approximately 83% of the giant panda locations from the 2014 Fourth National Giant Panda Census at Liziping were greater than 10.9 km from a residence, much farther away than would be predicted based on the available habitat within the reserve. This pattern was not seen in Daxiangling, where few dogs lived in the local villages and even fewer were free-roaming. The distribution of giant panda locations in Daxiangling did show a small shift away from residences (6.0 km vs. 7.8 km) consistent with the 1.92 km edge effect distance (due to humans alone) suggested by previous authors^35,36^. This shift was within one standard deviation of the mean of the available habitat.

### Extrapolation to range-wide impacts

**Figure 6:**
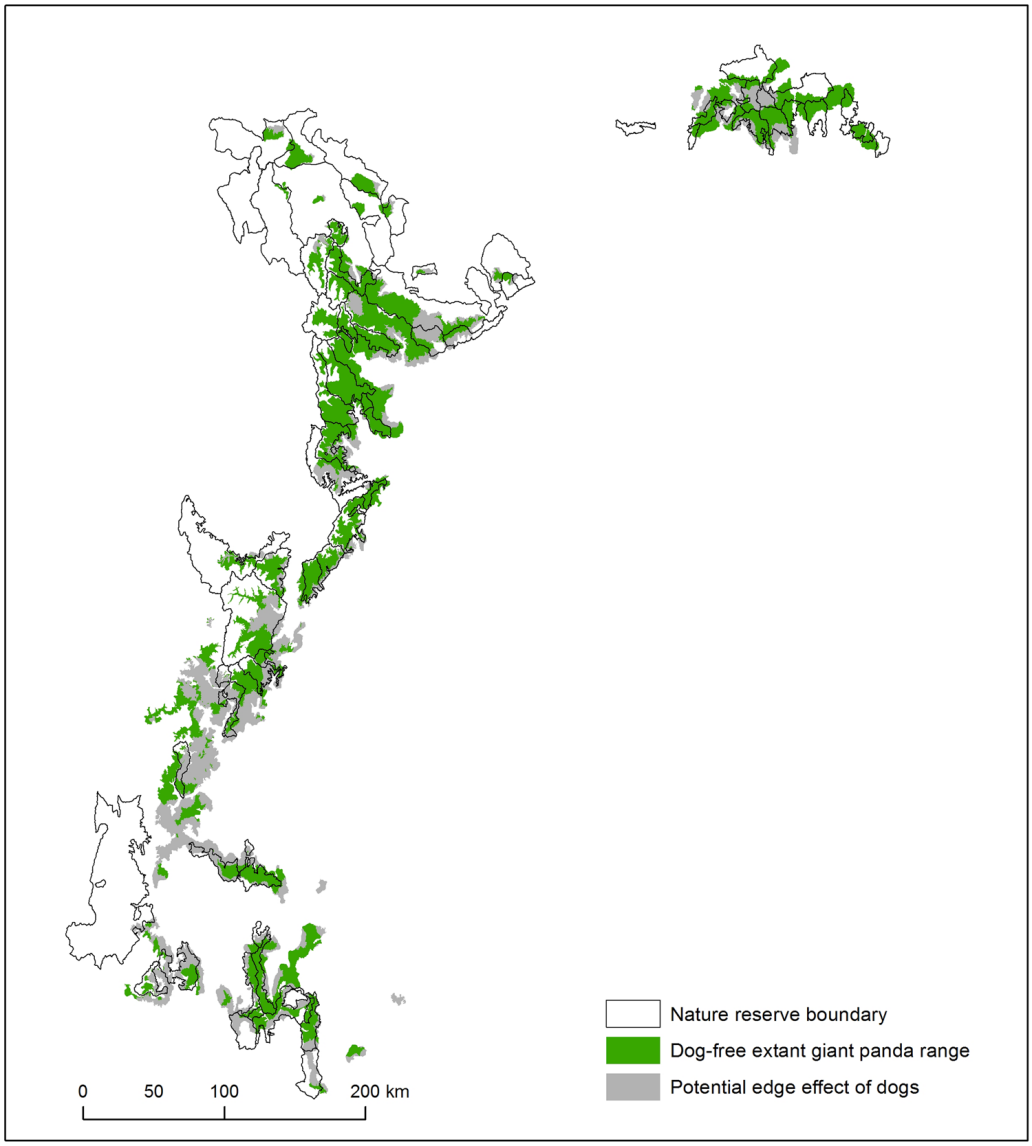
Range-wide assessment of potential edge effects of free-roaming dogs on giant pandas. Gray areas show extant giant panda range that is within the travel distance of free-roaming dogs (10.9 km path-distance). Green areas show potentially “dog free” giant panda range. Map produced using ArcGIS 10.6.1: https://desktop.arcgis.com/en/.

Range-wide analysis of potential edge effects of free-roaming dogs showed that 40% of the total giant panda range was within the foray distance of free-roaming dogs (Table 3). Giant panda range within reserves scored better with only 30% of habitat occurring within the foray distance of free-roaming dogs. However, edge effect was not distributed evenly across the geographic range of giant pandas (Fig. 6). The majority of edge effect was concentrated in the southern portion of the giant panda range with 6,730 km^2^ (66%) of the edge affected area south of the 31^st^ parallel. Gentler topography along the Dadu and Qingyi rivers in the south facilitated development and provided shorter path-distances to giant panda habitat. In the northern portion of the range, dramatic topographic relief, as well as lower human population density, reduced the vulnerability of giant panda populations to the threat of free-roaming dogs.

**Table 3:** Extent of potential edge effects of dogs on giant panda range inside and outside all 67 protected nature reserves.

	Total area	Edge effect area	Dog free area	Percent dog free
Extant giant panda range	25,710 km^2^	10,240 km^2^	15,470 km^2^	60%
Extant giant panda range in reserves	13,720 km^2^	4,060 km^2^	9,660 km^2^	70%

**Figure 7:**
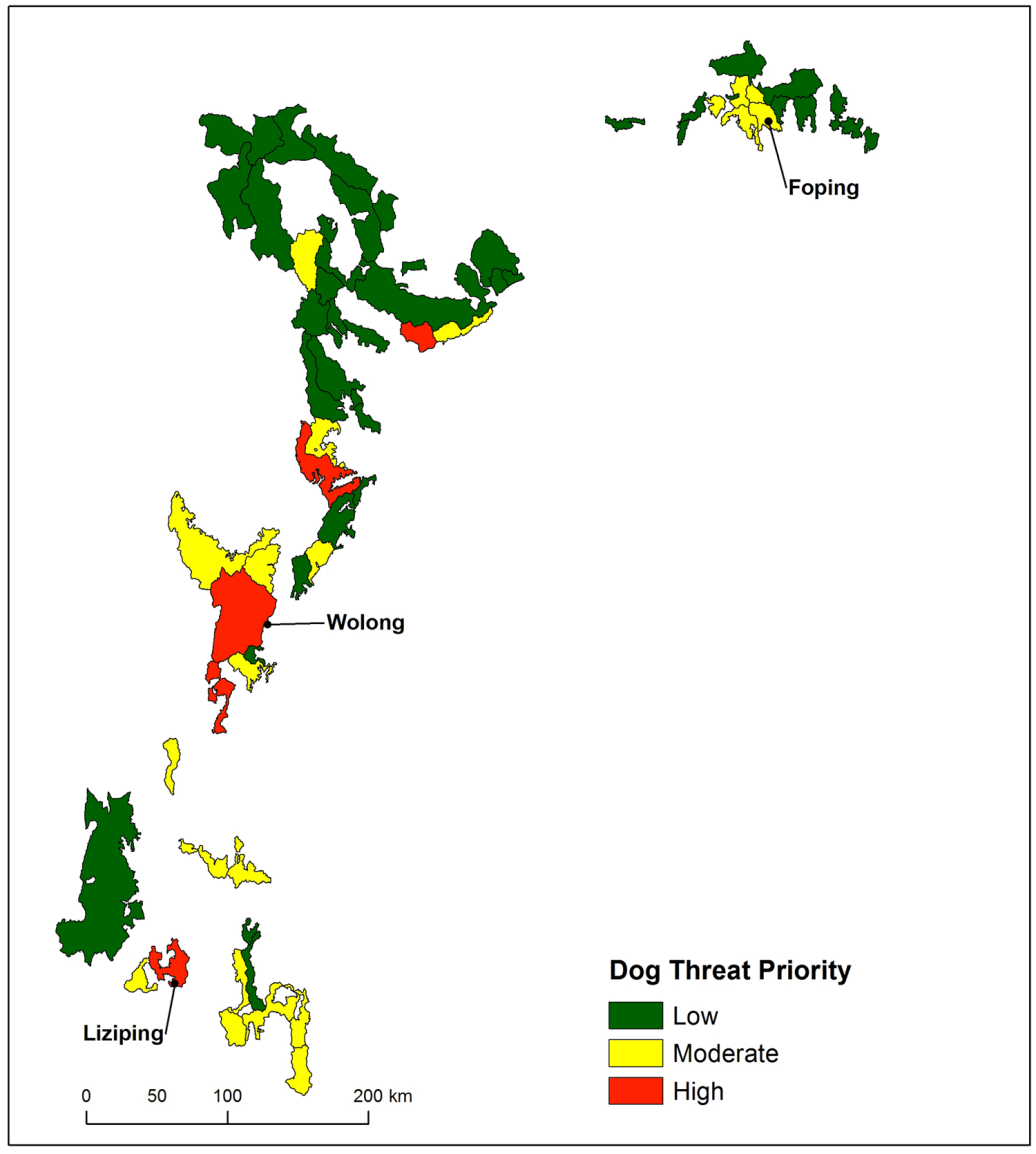
Giant panda nature reserves prioritized for dog control programs based on the area of panda habitat within 10.9 km path distance of human residences (Low < 50 km^2^, Moderate <165 km^2^, and High > 165 km^2^). Map produced using ArcGIS 10.6.1: https://desktop.arcgis.com/en/.

The amount of habitat within the dog zone in each reserve ranged from none (in several remote reserves) to 367 km^2^ (in Wolong Nature Reserve). Our classification of the giant panda nature reserves based on dog threat categories resulted in five nature reserves falling into the high risk category (including Liziping and Wolong Nature Reserves). Twenty-two of the reserves were classified as moderate while the majority of reserves (40 in total) received a low threat classification (Fig. 7).

## Discussion

Mapping the potential spatial extent of the edge effect of free-roaming dogs in giant panda habitat proved very informative. Without considering the threat of free-roaming dogs, Liziping Nature Reserve potentially provided more protected habitat for giant pandas than did Daxiangling Nature Reserve. However, Daxiangling had substantially fewer free-roaming dogs in villages and none observed within the reserve. In contrast, Liziping had hundreds of free-roaming dogs with easy access to the habitat within the reserve. The spatial pattern of giant panda distribution in Liziping was indicative of a risk effect (direct lethal, indirect lethal, and/or indirect non-lethal) in response to free-roaming dogs. The threat posed by free-roaming dogs has the potential to reduce the effective giant panda habitat by 72% at Liziping. In contrast, this was not the case in Daxiangling, where the spatial distribution of giant panda locations appeared to be in response to human impact alone (without dogs). Thus, we estimated that the available effective giant panda habitat at Daxiangling was 271 km^2^, much greater than the effective giant panda habitat in Liziping (109 km^2^). The larger total area occupied by the Liziping Nature Reserve (479 km^2^) was deceptive since the effective habitat area may be considerably smaller (109 km^2^).

The spatial clumping of giant pandas within the “dog free zone” suggests an edge effect of free-roaming dogs on habitat use by giant pandas in Liziping Nature Reserve. However, there are other potential explanations for this spatial pattern. For example, it is possible that the most highly suitable habitat for giant pandas is coincidentally found within the “dog-free zone.” There are also many additional threats to giant pandas originating from populated areas. Incidental take from poaching, bamboo harvesting, collection of medicinal plants and fuelwood collection have all been implicated as human factors influencing giant panda populations and habitat use^35,47^. According to Liu *et al*.^35^, however, effects from these disturbances are limited to a 1.92 km buffer around populated areas whereas our study suggests that the effect of free-roaming dogs extends to ~10.9 km path distance away from populated areas. Livestock use of giant panda habitat at Wanglang Nature Reserve was shown to reduce giant panda habitat by 34% but the distance from populated areas was not a factor measured in this study^48^. Impacts from livestock likely extend farther than the 1.92 km buffer distance cited for other human factors by Liu *et al*.^35^. The effect of livestock may be compensatory or additive when combined with the effect of free-roaming dogs.

A recent study estimated a carrying capacity of 163 individuals based on bamboo density (*Bashania spanostachya*) and high quality habitat in Liziping^42^. However, the existing giant panda population is a small fraction of this estimate, possibly due to the effective habitat area being much smaller than the suitable baseline habitat. If the issue of free-roaming dogs is addressed at Liziping, the effective giant panda habitat area should increase, simultaneously increasing the actual carrying capacity of the reserve.

The edge effect of dogs on giant panda habitat use is likely compounded by the spread of disease. In a recent study in our laboratory, 5% of surveyed dogs surrounding Liziping Nature Reserve tested positive for exposure to canine distemper virus and 13% tested positive for exposure to rabies virus^34^, both of which are potentially lethal for giant pandas. The importance of vaccinating and monitoring village dogs for canine distemper virus, canine coronavirus and rabies cannot be overstated. Dogs travel great distances (up to 35 km) when rabid^49^. At this effect distance, an outbreak of rabies would expose all individual giant pandas in a reserve to the disease threat.

Future studies should focus on the factors that influence dog foray distance. Does group size determine foray distance and influence “wolf-like” behaviors such as harassing and preying on wildlife? What role does size, age and gender of individual dogs play? Clearly, dog owners influence the likelihood of dogs entering nature reserves through variability in restriction methods. Owners may also unintentionally encourage forays by initially taking their dogs on resource extraction excursions into the reserve and/or by limiting the amount of supplemental food the dogs receive. We theorize that dog foray distance is a probabilistic variable that would best be modeled using a known distribution once enough spatial data is collected to set the parameters. Generating a surface of known probability of dog encounters would provide a more mechanistic understanding of this relationship and more clearly inform management efforts. For example, at what threshold of dog impact do giant pandas alter their habitat use? Do they respond to even low risks of encounters? Anecdotal evidence suggests that even solitary medium sized dogs will harass giant pandas but it is likely that only groups of dogs can inflict serious injuries. Solitary dogs still pose lethal risks to giant panda cubs and can introduce pathogens.

Based on our case studies in Liziping and Daxiangling, free-roaming dogs have the potential to limit giant panda habitat use, reproductive success and population persistence. To prioritize future studies and management efforts, we provided spatial data identifying areas potentially threatened by free-roaming dogs within the entire range of giant pandas (Fig. 6). Our basic spatial analysis is only a preliminary step. The resulting maps can be used as a tool for managers to select the most crucial nature reserves (shown in red in Fig. 7) for more extensive surveys of the local free-roaming dog populations. Where the distribution of free-roaming dogs and giant pandas overlap, we recommend the initiation of extensive GPS telemetry studies, as was recently performed in Foping Nature Reserve^51^. In Foping Nature Reserve (33.658°N 107.807°E), researchers tracked 85 dogs from two villages near the reserve, of which 41 traveled outside of the village in 8 packs^51^. This study did not indicate the total number of dogs nor the proportion that were free roaming in villages surrounding the reserve. However, given that nearly 50% of the monitored dogs left the village to enter giant panda habitat, it is clear that the dog problem in Liziping is not an isolated issue and the preliminary spatial analysis we present here is of value to other reserves in the network.

The study in Foping^51^ provides a template for future in-depth studies of free-roaming dog impacts on giant panda habitat use. We found that the majority of edge affected giant panda habitat was south of 31°N latitude and suggest that future studies be initiated in the southern mountain ranges which support the smallest and most isolated populations of giant pandas^28^. Giant pandas struggling to survive in this fragmented habitat are already susceptible to the suite of demographic and genetic threats facing small populations. Controlling free-roaming dog populations surrounding these southern reserves is a critical, but currently unutilized, management tool for preventing these small giant panda populations from going locally extinct.

As part of The National Conservation and Management Plan for the Giant Panda and its Habitat, the reintroduction of giant pandas has been initiated in two nature reserves (Wolong and Liziping). When selecting sites for translocations and reintroductions of giant pandas, there are many factors to consider: habitat availability and quality, the size and sex ratio of the existing population, bamboo species composition and abundance, connectivity to other giant panda populations, threats from competitors, disease and predators, road density and proximity to populated areas. The threats posed by local free-roaming dog populations must be added to the list of factors addressed for any comprehensive assessment aimed at selecting sites for conservation actions (particularly the release of naïve translocated individuals). Thus, these reserves should also receive top priority for camera traps and field surveys for dogs. Results indicating an effect of dogs on giant panda habitat use should trigger dog control programs focused on education for residents, free neuter and vaccination clinics and removal of feral dogs.

Where they are abundant and in close proximity to giant panda habitat, dogs are likely a greater threat to giant pandas than snares, bamboo harvesting, and natural predators. As such, free-roaming and feral dogs must be removed from nature reserves. Spay and neuter programs, as well as vaccination programs, should be implemented in all villages within 10 km of reserves. Comprehensive approaches that involve dog licensing and collaring need to be organized by local governments and implemented by village leaders. Local villagers are very supportive of giant panda conservation but do not realize that their dogs pose serious threats to giant pandas. In addition, the occupation and lifestyle of the residents influence the number of dogs per household and the level of control exerted over those dogs^52^. Education programs for local villagers that address the threat of free-roaming dogs are essential. Research shows that responsible management can greatly reduce the ecological impact of dogs in protected areas^50^.

Working with local governments can facilitate the implementation of novel solutions based on local needs. The employment of local villagers to help monitor the feral and free-roaming dog populations will improve the success of these programs. Consideration for the ethical treatment of dogs removed from reserves should be incorporated into all stages of the dog management plan through consultation with the Society for the Prevention of Cruelty to Animals or similar local groups. The dog population in China was estimated at 250 million in 2012 based on the human census and assumed human: dog ratios^11^. The threat these animals pose to giant pandas, the national treasure of China, should serve to galvanize control efforts. The creation of the Giant Panda National Park provides an opportunity to standardize and enforce dog control programs throughout the range of the giant panda.

Setting aside nature reserves for giant pandas has protected thousands of other species^53^. Addressing the dog problem in these reserves will also benefit biodiversity by protecting other vertebrate species from dog predation, harassment and disease transmission. Areas with feral dog populations have lower species diversity of terrestrial mammals and lower abundance of extant species^54^. The mountains of Sichuan are considered one of the world’s top biodiversity hotspots^55^. It is likely that free-roaming and feral dogs are a major unrecognized threat to the biodiversity of the region. In addition, China has the second highest incidence of human rabies cases^56^; therefore, controlling the dog population will also benefit public health for rural residents.

Our study illustrates that the effectiveness of nature reserves depends upon our understanding and management of complex interactions between the still increasing populations of humans and their domestic animals and the wild animals such as giant pandas that we are trying to protect. While widely acknowledged by ecologists, edge effects at broad spatial scales are rarely addressed explicitly when tackling the numerous threats facing species of concern. Recent studies of the edge effects of intrusive free-roaming dog populations on the distribution of threatened species in nature reserves^23,27,51^ indicate a need for comprehensive changes to policy for managing biodiversity in protected areas. Only through the development and application of this knowledge can we hope to sustain natural systems in a world increasingly crowded by humans.

## Data Availability

The locations of human residences surrounding Liziping and Daxiangling Nature Reserves as well as populated areas for the entire giant panda range will be made available on http://databasin.org/datasets/ upon acceptance of the manuscript. The 2014 National Giant Panda Census data are sensitive, protected by the State Forestry Administration, P.R.C., and are not generally available. We only had access to the giant panda census data for the two reserves that we studied in detail (Liziping and Daxiangling).
